# Sonoelastography - A new paradigm in the characterization of breast lesions

**DOI:** 10.6026/973206300214610

**Published:** 2025-12-15

**Authors:** Zaryab M Qureshi, Kavita U Vaishnav, Rutvik G Patel, Harsh A Jagetiya, Jay M Chaudhary, Burhanuddin F Padrawala, Maitry R Talavia, Hetavi B Patel

**Affiliations:** 1 Department of Radiology, Narendra Modi Medical College and L.G. Hospital, Ahmedabad, Gujarat, India

**Keywords:** Sonoelastography, breast lesions, diagnostic imaging, b-mode ultrasound, sonomammography, elastography

## Abstract

The diagnostic performance of sonoelastography compared to conventional sonomammography in 136 patients with palpable breast lesions at L.G. Hospital, Ahmedabad is of interest. Hence, patients underwent B-mode ultrasound, mammography, and real-time elastography, with histopathology as the reference standard. Sonoelastography demonstrated higher specificity (90% vs. 60%), PPV (87% vs. 64%), and overall diagnostic accuracy (82%) compared with sonomammography, while maintaining comparable sensitivity (93.3%). The addition of elastography reclassified 30% of benign lesions correctly, reducing unnecessary biopsies and preserving 100% sensitivity in high-risk BIRADS categories. Thus, we show that sonoelastography is a valuable complementary tool to sonomammography in breast lesion characterization.

## Background:

Mammography and ultrasonography (US) are the chief screening modalities which have shown the highest sensitivity in the early detection of breast cancer to reduce mortality rates. However, both methods have their own limitations. Mammography shows decrease in sensitivity in dense breast tissues and often yield false-negative results. Breast US has poor ability to distinguish isoechoic lesions from surrounding fat, inability to image areas deep within the breast, poor detection of microcalcifications and poor specificity as most solid lesions turn out to be benign. The BI-RADS criteria generate a significant number of false positive results. This limitation leads to increased biopsies performed in benign lesions causing discomfort to the patients with a cancer -detection rate || of only 10%-30%. To overcome these limitations and obtain a more accurate way to characterize breast lesions, US elastography was introduced in 1990. It is a new imaging technique that identifies the elasticity of the tissue (stiffness) *in vivo* [1]. It is an imaging equivalent of clinical palpation. Elasticity is the property of a tissue that enables it to be deformed when it is subjected to an external force and resume its original shape or size when the force is removed. Tissue deformation is inversely proportional to the stiffness of the material, and response time (i.e. return to the natural condition). Two main types of elastography, strain(SE) and shear-wave (SWE), are commonly used to image breast tissue. SE causes displacement of tissue when compression is applied. Strain is exhibited by breathing, contraction, external compression by probe or push pulse. Strain elastography measures relative stiffness of lesion compared to normal breast parenchyma SWE works with the acoustic radiation force excitation and real-time shear velocity (RSV) to display an image of the shear-wave speed. Shear wave elastography measures absolute stiffness of the lesion. Many studies have reported that ultrasound elastography can increase the specificity of mammosonography in differentiating benign from malignant breast masses [2]. Recently, the 2nd edition of the Breast Imaging Reporting and Data System (BI-RADS) US lexicon also has added the US elastographic features of breast masses as an associated finding. However, this technique is still new; its role in clinical practice is still to be defined and therefore, it is of interest to show how does elastography helps for the same [3].

## Methods and Materials:

Source of Data: Patients presenting to L.G. hospital, Maninagar, Ahmedabad, with complaint of breast lesions from period of March 2023 to July 2025. They were further referred to Radiology Department.

## Study design:

A retrospective record based study. All patients were subjected to B-mode Ultrasound, mammography and sonoelastography. Histopathological results were taken as gold standard (Figure 4 - see PDF). Patient population 136 patients of age groups 20 to 70 years were evaluated. Equipment and procedure: GE APLHA ST mammography system (MLO and CC views taken) PHILIPS 70 AFFINITI US Machine equipped with real time elastography software and Linear probe with bandwidth of 3-12 MHz for B mode ultrasound and elastography was used for US and elastography.

## Measurements:

[1] Various parameters of the suspicious breast lesion on USG and mammography were recorded and accordingly BIRADS category was assigned using the BIRADS classification by the American College of Radiology (2013).

[2] Strain Elastography was performed with slight probe pressure over the region of interest and lesions were graded according to the elastography scoring system (Tsukuba score) and Elastography Strain ratio. The elastogram, is created as a colour coded map (the areas of great stiffness are coded in RED, those which are more deformable in BLUE, and green indicates intermediate levels of elasticity).

[3] Cut off of >2.5 was decided to distinguish between benign and malignant lesions (Figure 3 - see PDF).

[4] Statistical analysis was performed by calculating sensitivity, specificity, PPV and NPV for each, sonomammography, Tsukuba score and strain ratio.

## Results:

Lesions are most common in the 31-40 age groups. Benign lesions decrease with age; malignant lesions peak in 41-50.

## Diagnostic Performance (vs histopathology):

Sensitivity 93.3% | Specificity 60% | PPV 64% | NPV 92% | Accuracy 74%

Sensitivity 93.3% | Specificity 90% | PPV 87% | NPV 94% | Accuracy 82%

Sensitivity 93% | Specificity 85% | PPV 82% | NPV 94% | Accuracy 88%

## Statistical comparison and significance:

[1] Mammosonography: p = 0.0015

[2] Elastography (Tsukuba): p = 7.07 x 10^-7^

[3] Strain Ratio: p = 4.06 x 10^-6^

Compared to sensitivity of 60 % on mammosonography, elastography shows higher specificity of 85-90%. PPV and NPV also increase in elastography.

## Case of Intraductal Papilloma:

[1] Complain of: Left breast pain since 2 months.

[2] Mammography : As shown in Figure (6A - see PDF), Dilated radiolucent duct in retroareolar region of left breast

[3] Ultrasonography: As shown in Figure (6B - see PDF), Single dilated duct with few intraductal echogenic lesions in left breast.

[4] BIRADS- 3, Tsukuba score- 3, Strain Ratio-1.16 Figure (6C - see PDF) (Table 5-6 - see PDF).

## P-values and 95% confidence intervals(Fisher's exact test):

[1] Mammosonography: p = 0.0015

[2] Elastography (Tsukuba): p = 7.07 x 10^-7^

[3] Strain Ratio: p = 4.06 x 10^-6^

95% confidence intervals (CI)- Sensitivity CI and Specificity CI for different methods:

## Interpretation:

[1] Mammosonography has a lower specificity and a wider confidence interval for specificity.

[2] Elastography (Tsukuba Score and Strain Ratio) significantly improve specificity (higher CI).

[3] Elastography is statistically superior to mammosonography (p-values < 0.0001), confirming its higher diagnostic accuracy.

## Case of Complex Fibroadenoma:

[1] Complain of: Left breast lump since 2 months (Table 2 - see PDF).

[2] Mammography: As shown in Figure (5A - see PDF), a well-defined equal density lesion at 12 o'clock position with surrounding radiolucent halo in central and retroareolar region.

[3] Ultrasonography: As shown in Figure (5B - see PDF), a well-defined iso to hypoechoic lobulated lesion, p/o ? Atypical fibroadenoma or pseudoangiomatous stromal hyperplasia.

[4] BIRADS- 3, Tsukoba score-2, Strain ratio- 0.56 Figure (5C - see PDF) (Table 5-6 - see PDF).

[5] Histopathology: Benign proliferative lesion, s/o Complex Fibroadenoma.

## Case of invasive ductal carcinoma:

[1] Complain of: Left breast lump since 8 years (Table 2 - see PDF).

[2] Mammography: As shown in Figure (7A - see PDF), Well defined high density lesion with microlobulated margins and internal pleomorphic microcalcifications and spiculated margins towards nipple.

## Case of Duct Ectasia:

[1] Complain of: Swelling in right breast since 1month with nipple discharge, gradually increasing in size.

[2] Mammography: As shown in Figure (8A - see PDF), Malignant mass lesion in retroareolar region with metastatic axillary lymphadenopathy.

[3] Ultrasonography: As shown in Figure (8B - see PDF), Taller as wider ill-defined hypoechoic lesion in upper outer quadrant of right breast with dilated ducts without any vascularity.

[4] BIRADS 4, Tsukuba Score: 2, Strain ratio: 1.69 Figure (8B - see PDF) (Table 5-6 - see PDF).

[5] Histopathology: Benign lesion, suggestive of Duct Ectasia.

## Case of Giant Fibroadenoma:

[1] Complain of: Painless, large, freely movable lump since few months (Table 2 - see PDF)

[2] Mammosonography: As shown in Figure (9A - see PDF), Well defined equal density lesion in retroareolar region.

[3] Ultrasonography: As shown in Figure (9B - see PDF), Well defined lesion with lobulated margins and posterior acoustic enhancement.

[4] BIRADS- 3, Tsukuba score- 2, Elastography ratio- 0.46 Figure (9C - see PDF) (Table 5-6 - see PDF).

[5] Histopathology- Benign lesion, suggestive of- Giant fibroadenoma.

A total of 136 female patients were included, mostly aged 41-50 years. Clinically, the majority presented with a lump (Table 2 - see PDF). All patients were female. Imaging BIRADS categories 2-5 were most common, with BIRADS 5 predominating. Mammosonography showed 93.3% sensitivity and 60% specificity. Elastography with Tsukuba scoring improved specificity to 90% with similar sensitivity (Table 5, 8 - see PDF), while strain ratio (>2.5) showed 93% sensitivity and 85% specificity (Table 6, 8 - see PDF). Statistical analysis confirmed significantly better diagnostic performance for elastography compared to mammosonography (p ≤ 7.07 x 10^-7^) (Table 8 - see PDF), with confidence intervals supporting higher specificity for elastography.

## Discussion:

In this study of 136 patients, the significant clinical impact of integrating sonoelastography into breast lesion assessment is evident. The demographic pattern, with benign lesions predominantly in the 31-40 age group and malignancies peaking in the 41-50 age groups (Table 1 - see PDF), mirrors epidemiological data and underscores the influence of hormonal, age-related risk factors and sex related (Table 3 - see PDF) [4]. While both sonoelastography and mammosonography demonstrated high sensitivity (~93%), sonoelastography markedly enhanced specificity, rising from 60% with conventional imaging to 90% using the Tsukuba score and 85% with strain ratio (Table 7 - see PDF and Figure 2 - see PDF). This improvement translated into higher PPV (87% vs. 64%), NPV (94% vs. 92%), and overall accuracy (82% vs. 74%), thereby equipping clinicians with more reliable diagnostic tool. A key clinical finding is the ability of sonoelastography to accurately downgrade low-suspicion lesions (BIRADS 3 and 4A), potentially avoiding biopsies in at least 15 patients (Table 4 - see PDF). This aligns with broader research showing that elasticity imaging reduces benign biopsy rates by 30-40%, optimizes patient management, alleviates anxiety, and lowers healthcare costs [5]. Quantitative tools like Virtual Touch IQ have demonstrated reductions of up to 15% in benign biopsies while maintaining diagnostic yield, suggesting that integrating elastography reduces overtreatment without missing malignancies. BI-RADS 3 and 4 with benign elastography features could be safely downgraded, reducing false-positive malignancy calls and biopsies [5]. Sonoelastography also adds value in cases where B-mode ultrasound is equivocal, especially in dense breast tissue where mammography underperforms, as quantitative stiffness data enhances BI-RADS classification and improves triage decisions. Sonoelastography showed higher sensitivity (91.7% at strain ratio cutoff ~3) for detecting malignant breast lesions than both mammography (87.9%) and conventional US (90.9%) [6]. Despite these advantages, sonoelastography remains sensitive to technique, particularly in strain elastography, where operator pressure affects reliability. Interpretation variability also persists across operators. Standardized protocols, adoption of shear-wave techniques, and advances in AI-driven automation may mitigate these challenges by providing quantitative and reproducible metrics. Although the expanded cohort improves analytical power over earlier studies, this remains a single-center study. Multi-institutional research is needed to confirm generalizability and establish universal elastographic benchmarks. Future work comparing strain versus shear-wave techniques and longitudinal studies evaluating elastography in treatment monitoring and recurrence prediction will further refine its clinical role. Sonoelastography, as an adjunct to conventional breast imaging, improves specificity, PPV, NPV, and diagnostic accuracy while maintaining high sensitivity [4, 7]. Importantly, it enables confident differentiation of low-suspicion lesions and reduces unnecessary biopsies. While observer dependence remains a limitation, the potential benefits in reducing patient burden and optimizing resource utilization strongly support its integration into routine breast imaging protocols. Future multi-center and standardized studies will be pivotal in consolidating its role in clinical practice [8].
